# Oil of Codfish in Scrofulous Diseases

**Published:** 1840-07

**Authors:** 


*On the employment of the Oil of Cod Fish in Scrofulous Diseases.* By M. Taufflied.—[The beneficial influence of the oil of cod fish in certain forms of scrofulous disease, has within the last few years been dwelt on by various German writers; M. Taufflied reports eight cases, from which he draws the following inferences confirmative of their accounts.] 1. The oil of cod fish exercises a favorable influence on the general state of lymphatic subjects. 2. If administered with proper care it possesses the property of curing scrofula of the bones, tabes mesenterica, and scrofulous or rheumatismal chronic arthritis. 3. Caries accompanied with solution of continuity and engorgement of the soft parts requires local treatment in addition to the oil administered internally. Under these circumstances compression and alcoholic iodu-retted fomentations may be successfully employed. 4. The oil of cod fish has no efficacy in cases of gouty arthritis, nor does it exercise any influence on the engorgement of any lymphatic glands except the abdominal. It seems to have little or no effect on scrofulous phthisis, if this be at all advanced. 5. The oil should be administered perseveringly and for several months, in order to secure advantageous results.—*Gaz. Medicale de Paris.*—*Bost. Med. and Surg. Jour.*

